# Comparison of pregnancy outcome in intrauterine insemination-candidate women with and without endometrial scratch injury: An RCT

**DOI:** 10.18502/ijrm.v19i5.9255

**Published:** 2021-06-23

**Authors:** Mahnaz Yavangi, Nesa Varmaghani, Azar Pirdehghan, Maryam Varmaghani, Mohammad Faryadras

**Affiliations:** ^1^Endometrium and Endometriosis Research Center, Fatemieh Hospital, Hamadan University of Medical Sciences, Hamadan, Iran.; ^2^Department of Obstetrics and Gynecology, School of Medicine, Hamadan University of Medical Sciences, Hamadan, Iran.; ^3^Department of Community and Preventive Medicine, School of Public Health and Research Center for Health Sciences, Hamadan University of Medical Sciences, Hamadan, Iran.; ^4^Department of Radiology Medicine, School of Public Health and Research Center for Health Sciences, Hamadan University of Medical Sciences, Hamadan, Iran.; ^5^Department of Epidemiology and Biostatical, School of Public Health, Hamadan University of Medical Sciences, Hamadan, Iran.

**Keywords:** Pregnancy infertility, Women, Endometrial injury, Pregnancy, Intrauterine insemination.

## Abstract

**Background:**

Endometrial scratch injury is considered controversial in increasing the success rate of assisted reproductive technology.

**Objective:**

To compare the pregnancy outcomes in women undergoing intrauterine insemination with and without an endometrial scratch.

**Materials and Methods:**

In this randomized clinical trial, 150 women referred to the Fatemieh Hospital, Hamadan, Iran who were candidates for IUI between December 2017 and December 2018 were randomly assigned into two groups (n = 75/each) with or without an endometrial scratch (as case and control groups, respectively). Women in both groups were in proper and identical protocol for IUI. Chemical and clinical pregnancies, abortion, and live birth rate, also pregnancy complications were compared between the groups.

**Results:**

Chemical and clinical pregnancy rates were higher in the case than the control group (p = 0.25, p = 0.54, respectively). In the case group, the abortion and multiple gestation rates were 14.3% and 4.3%, respectively, while it was 5% in the control group (p = 0.60, p = 0.54 respectively). The endometrium thickness on day 21 was higher in the case group than the control (p = 0.01).

**Conclusion:**

Endometrial scratching in intrauterine insemination women is not associated with an increase in both clinical and clinical pregnancy rates, however, studies with a larger sample size are recommended to evaluate this intervention.

## 1. Introduction

Intrauterine insemination (IUI) involves a variety of procedures, all done by placing whole or prepared sperm into the uterine cavity (1). The success rate of IUI depends on several factors, including IUI time, catheter type, ovulation induction method, inoculated semen volume, and inoculation (2). IUI can be used to normalize the ovulation cycle, but nowadays, ovulation stimulation cycles are used more often for infertility treatment because they make the timing of the procedure more accurate and the timing of ovulation more predictable (3).

Embryo implantation is one of the most important causes of the failure of assisted reproductive methods, including IUI (4). The IUI time is determined by considering the number and diameter of the follicles and the size of the endometrial thickness to obtain the best results. While some studies have shown that itching or intentional injury to the endometrium results in better outcomes, others have found no difference and have reported no beneficial results (5–7). Some studies that have found beneficial results of endometrial scratching are studies with small sample sizes that are not reliable (8, 9). A successful pregnancy requires good-quality oocyte and sperms that lead to a good-quality embryo and an endometrium that is ready for the embryo. Generally, days 19 to 23 of the menstrual cycle are the thickest the endometrium remains ready for implantation (implantation window).

Scratch or damage to the endometrium is a simple and inexpensive procedure that can be performed outpatient without the need for anesthesia. This procedure can be performed with a Novak catheter or through Pipelle biopsy (endotracheal tube) (10, 11).

However, whether endometrial scratching is beneficial for women undergoing IUI still remains controversial and cannot be answered with certainty. Given the small number of national studies on the impact of endometrial scratching on pregnancy outcome, especially in women undergoing IUI, the controversial results of previous studies, and the high cost of other methods such as in vitro fertilization and its potential risks, the aim of this clinical trial study was to compare the pregnancy outcomes in women undergoing IUI with and without endometrial scratching.

## 2. Materials and Methods

Out of the 300 estimated sample size, 150 cases were removed due to lack of financial resources and time and the study continued with 150 participants. This study is a single blind randomized clinical trial and included 150 eligible women referred to the obstetrics and gynecology ward of the Fatemieh Hospital, Hamadan, Iran from December 2017 to December 2018. Women were randomly divided into two groups of with (case) and without (control) endometrial scratching using a random number table. Patients were blind to the type of intervention. Therefore, the trial was run as a single blind. The inclusion criteria were: age 18–40 yr, infertility, body mass index ≤ 30 and >18, normal menstrual period and normal fallopian tube. The exclusion criteria were hirsutism, autoimmune disease, endocrine disease, smoking and alcohol intake. For all the participants' partners, sperm analysis was performed to determine the cause of infertility. The levels of thyroid-stimulating hormone (TSH), follicle-stimulating hormone (FSH), luteinizing hormone (LH), and prolactin were measured in all women. All laboratory tests were performed at the hospital lab and all kits were similar (Chemiluminescenc, Germany). Laparoscopic hysterosalpingogram were performed to diagnose uterine and tubal causes.

Clomiphene citrate (OOVOMID, Iran-Hormone) and human menopausal gonadotropin (HMG) (Ronak Pharmaceutical Co., Iran) were prescribed for ovulation induction before initiating the IUI. All women on the third day of the cycle following a transvaginal ultrasound received clomiphene 100 mg (Hormone, Iran) daily for five days and HMG 75 IU was injected intramuscularly on days 6 and 7. The women were followed-up again on days 11 or 12 of the cycles through vaginal ultrasonography followed by at least 18-mm follicle in the ovary. The 5000-unit human chorionic gonadotropin (HCG) (Hormone, Iran) was injected intramuscularly and 36 hr later, 0.5 ml of washed sperm was inserted after catheter and lithotomic preparation, the cervical catheter was then gently inserted into the uterus. For luteal-phase support, 50 mg progesterone (Hormone, Iran) was administered daily for up to 2 wk.

Women were advised to undergo endometrial scratching on days 19 to 21 of their cycle by the treating physician. Considering the high chances of implantation as the endometrial scratching causes an inflammation processes in the endometrial layer creating a proper condition for implantation in an IUI cycle (12). Women were also advised not to have intercourses beforehand.

Endometrial scratching was done by Pipelle (Medbar Company, USA) in sterile conditions and a lithotomic position in four directions (12–6–9–12 hr). Endometrial thickness was measured on days 19 to 21 of the cycle in all women. In addition, women were given two doses of 500 mg azithromycin as prophylaxis. The chemical and clinical pregnancies as well as the endometrial thickness were measured.

### Ethical considerations

This study was approved by the ethics committee of the Hamadan University of Medical Sciences, Hamadan, Iran (Code: IR.UMSHA.REC.1396.153). In addition, the study proposal was registered at the Iranian Registry of Clinical Trials. The participants were informed about normal infertility treatments and IUI processes and study protocol, after which they signed an informed written consent.

### Statistical analysis

Data were analyzed using the Statistical Package for Social Sciences (SPSS Inc., Chicago, IL, USA). Data were expressed using descriptive statistics with mean and standard deviation (SD) for quantitative variables and ratio and percentage for qualitative variables. A Chi-square test was used to compare the relationship between the qualitative variables and student's *t* test or non-parametric equivalent was used to compare the quantitative variables. P-value < 0.05 was considered as statistically significant.

## 3. Results

**Figure 1:**
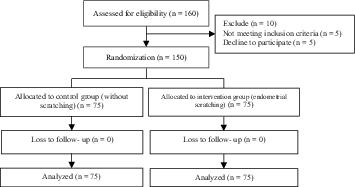
The consort intervention flowchart of the study.

In this clinical trial study, 150 infertile women undergoing IUI who met our inclusion criteria were randomly assigned into two groups (n = 75/each). Figure 1 shows how patients were selected and followed-up. Both groups were compared in terms of the mean age, duration of infertility, body mass index, number of dominant follicles, type of infertility, cause of infertility, and history of IUI (Table I). The numbers, motility, and morphology of semen fluid prepared for intrauterine injection were compared in both groups (Table II).

A significant increase was seen in the endometrial thickness in the case group (approximately 1.5 mm) (9.6 ± 1.2 vs 8.1 ± 2.0, p < 0.001). In addition, pregnancy rate was 8% higher in the case group [21 (28%) vs 15 (20%)] but there was no statistically significant difference between the two groups (p = 0.251). The clinical pregnancy rate was also higher in the case group than in the control group [17 (22.7) % vs 14 (18.7%)] but no significant difference was observed (p = 0.545). Moreover, there was no significant difference between the case and control groups in the rate of miscarriage (14.3 % vs 5%, p = 0.606, respectively) and multi-gestation (4.8% vs 5%, p = 0.545, respectively) in women with positive pregnancy. In this study, the endometrial thickness was compared between the control and case groups in the previous IUI and the second IUI in this study with endometrial scratching (9.5 ± 3.6 vs 7.7 ± 3.4, p = 0.002).

**Table 1:** Comparison of demographic and baseline variables in the case and control groups


**Variable**		**Control group (n = 75)**	**Case group (n = 75)**	**P-value**
Age (yr)${}^{*}$		27.1 ± 5.1	26.9 ± 5.1	0.77
Infertility duration (yr)${}^{*}$		3.8 ± 2.1	3.7 ± 2.2	0.80
Body mass index (kg/m${}^{2}$)${}^{*}$		26.9 ± 1.8	27.4 ± 1.8	0.15
Dominant follicle in both ovaries${}^{*}$		2.5 ± 0.9	2.8 ± 1.1	0.07
Infertility type**				
**Primary**		62 (82.7)	65 (86.7)	0.49
**Secondary**		13 (17.3)	10 (13.3)	
Cause of infertility${}^{**}$				
	**Male factors**	26 (34.7)	29 (38.7)	
	**Unexplained**	17 (22.7)	12 (16.0)	
	**Female factors**	25 (33.3)	23 (30.7)	0.53
	**Male and female factors**	7 (9.3)	11 (14.6)	
Previous IUI history${}^{**}$		54 (72.0)	61 (81.3)	0.17
*Data presented as Mean ± SD *t* test, **Data presented as n (%), Chi-square test, IUI: Intrauterine insemination				

**Table 2:** Comparison of injected semen fluid parameters for uterine fertilization


**Parameter**	**Control group**	**Case group**	**P-value**
**Ml number (10${}^{6}$)**	37.8 ± 3.9	38.1 ± 4.5	0.658
**Morphology percentage**	61.7 ± 7.3	60.7 ± 8.9	0.452
**Motility percentage**	66.9 ± 19.9	70.8 ± 14.4	0.166
Data presented as Mean ± SD *t* test. MI: Milliliter			

## 4. Discussion

This clinical trial was performed to investigate the effect of endometrial scratching on women who were candidates for IUI on their pregnancy outcomes and its associated complications.

The findings of this study showed that endometrial scratching was associated with an 8% and 4% increase in chemical and clinical pregnancy rates, respectively, in the case group than in the control group, although this difference was not statistically significant but may be clinically significant (for patients and physicians). However, the observed difference could be statistically significant if the sample size was higher. Endometrial thickness was associated with a significant increase in the case group, which may indicate a better efficacy of this case in these women. IUI is an inexpensive, noninvasive method that is widely used in treating infertile couples. This method, along with controlled ovarian hyperstimulation, is widely used to treat infertility with cervical causes, male factor, ovulation disorder, mild endometriosis, which are not involved in fallopian tubes, and infertility of unknown cause (3, 7).

However, the success rate of IUI is estimated to be 10–25% (13). So, it is pertinent to work toward improving its success rate. Some studies have shown that endometrial scratching is associated with increased rates of pregnancy such as normal saline infusion, curettage, and hysteroscopy (7, 9, 14).

However, the results of the studies are controversial. Initially, endometrial scratching was performed in animal studies, which showed that scratching or trauma to the endometrial was associated with increased decidualization stimulation (15). Endometrial scratching is inexpensive and can be performed without the need for anesthesia; it can be beneficial in women in need of ART if performed properly. However, studies regarding endometrial scratching and IUI are limited. A study by Zarei and colleagues showed that endometrial scratching in women undergoing IUI was although not associated with a significant increase in clinical pregnancy rates per cycle and per person, it was associated with increased endometrial thickness and decreased estradiol (5). These findings are in line with the results of the present study. Similarly, El-Khayat and coworkers concluded that endometrial scratching in women with IUI candidacy was not associated with a significant increase in clinical pregnancy (4).

However, Karimzade and colleagues showed that endometrial scratching had a negative impact on pregnancy outcome (12). The findings of Karimzadeh et al. showed that the implantation rate (7.9% vs 22.9%), clinical pregnancy (12.3% vs 32.9%), and ongoing pregnancy (9.6% vs 29.1%) was significantly lower in the intervention group. A clinical trial showed that endometrial scratching with normal saline on days 3–5 of the menstrual cycle was not associated with beneficial outcomes in in vitro fertilization women with repeated implantation failure. Karimzadeh and colleagues showed that scratching with the luteal-phase biopsy in the previous cycle was associated with a significant increase in clinical pregnancy rate (27.1% vs 8.9%) (8). In a meta-analysis study, Nastri and colleagues reported a 2015 scratch with beneficial results in ART women (7).

It seems that one of the reasons for the differences in the results of the studies was the different times for endometrial scratching in the women. Studies in luteal-phase scratching seem to be associated with better outcomes in women (16). In fact, not knowing the timing of endometrial scratching and use of proper method for pipelle are limitations. We also used the perfect catheter in this study while some previous studies have used biopsy or some other methods, and this might have affected the final results. However, clarifying this issue will require further studies to increase the success of endometrial scratching and decision-making. It is yet not clear why endometrial scratching is associated with increased pregnancy rates. One of the possible causes is the induction of decidualization (17) and increased secretion of interleukins and growth factors, which is associated with increased endometrial acceptance (18, 19).

## 5. Conclusion

According to the findings of the present study, endometrial scratching in IUI-candidate women is not associated with an increase in chemical and clinical pregnancy rates. Further studies with larger sample sizes are recommended to confirm or reject this intervention.

##  Conflict of Interest

The authors declare that there is no conflict of interest.
